# Exocytosis of large-diameter lysosomes mediates interferon γ-induced relocation of MHC class II molecules toward the surface of astrocytes

**DOI:** 10.1007/s00018-019-03350-8

**Published:** 2019-10-30

**Authors:** Mićo Božić, Alexei Verkhratsky, Robert Zorec, Matjaž Stenovec

**Affiliations:** 1 Celica Biomedical, Tehnološki park 24, 1000 Ljubljana, Slovenia; 2 Laboratory of Neuroendocrinology-Molecular Cell Physiology, Institute of Pathophysiology, Faculty of Medicine, University of Ljubljana, Zaloška 4, 1000 Ljubljana, Slovenia; 3 Faculty of Biology, Medicine and Health, The University of Manchester, Manchester, M13 9PT UK; 4 Achucarro Center for Neuroscience, IKERBASQUE, 48011 Bilbao, Spain

**Keywords:** Astroglia, Inflammatory cytokines, Adaptive immunity, Patch clamp, Lysosomes, Fusion pore

## Abstract

Astrocytes are the key homeostatic cells in the central nervous system; initiation of reactive astrogliosis contributes to neuroinflammation. Pro-inflammatory cytokine interferon γ (IFNγ) induces the expression of the major histocompatibility complex class II (MHCII) molecules, involved in antigen presentation in reactive astrocytes. The pathway for MHCII delivery to the astrocyte plasma membrane, where MHCII present antigens, is unknown. Rat astrocytes in culture and in organotypic slices were exposed to IFNγ to induce reactive astrogliosis. Astrocytes were probed with optophysiologic tools to investigate subcellular localization of immunolabeled MHCII, and with electrophysiology to characterize interactions of single vesicles with the plasmalemma. In culture and in organotypic slices, IFNγ augmented the astrocytic expression of MHCII, which prominently co-localized with lysosomal marker LAMP1-EGFP, modestly co-localized with Rab7, and did not co-localize with endosomal markers Rab4A, EEA1, and TPC1. MHCII lysosomal localization was corroborated by treatment with the lysosomolytic agent glycyl-l-phenylalanine-β-naphthylamide, which reduced the number of MHCII-positive vesicles. The surface presence of MHCII was revealed by immunolabeling of live non-permeabilized cells. In IFNγ-treated astrocytes, an increased fraction of large-diameter exocytotic vesicles (lysosome-like vesicles) with prolonged fusion pore dwell time and larger pore conductance was recorded, whereas the rate of endocytosis was decreased. Stimulation with ATP, which triggers cytosolic calcium signaling, increased the frequency of exocytotic events, whereas the frequency of full endocytosis was further reduced. In IFNγ-treated astrocytes, MHCII-linked antigen surface presentation is mediated by increased lysosomal exocytosis, whereas surface retention of antigens is prolonged by concomitant inhibition of endocytosis.

**Electronic supplementary material:**

The online version of this article (10.1007/s00018-019-03350-8) contains supplementary material, which is available to authorized users.

## Introduction

Astrocytes, a class of morphologically and functionally heterogeneous neuroglial cells maintain homeostasis at all levels of organization of the central nervous system (CNS). Astroglial cells regulate transport of water and ions, provide metabolic support, participate in neurotransmission and synaptic connectivity, regulate microcirculation, and help preserve the integrity of the blood–brain barrier [1]. Astrocytes also contribute to defensive responses to CNS damage through a process of reactive astrogliosis [2–4]. Reactive astrogliosis is represented by a spectrum of morphologic and functional changes emerging in response to CNS injury and disease. It is regulated in a context-specific manner and promotes either gain or loss of function of astrocytes [2], thus influencing pathologic progression [5, 6].

Several inflammatory cytokines can instigate astrocyte activation and are, therefore, used to generate and analyze this process [3]. Interferon γ (IFNγ or type II interferon) is produced primarily by immune cells, such as activated T helper (Th) 1 and natural killer cells, and is traditionally regarded as a pro-inflammatory cytokine. Upon binding to IFNγ receptor 1 (IFNγ-R1), IFNγ stimulates the JAK/STAT signaling cascade and the consequent transcriptional response [7]. In astrocytes, IFNγ signaling appears to have a net pro-inflammatory effect [8, 9]. In professional antigen-presenting immune cells, such as dendritic cells, IFNγ increases the expression of major histocompatibility class II (MHCII) molecules. Exposure to IFNγ also induces expression of MHCII molecules in non-professional antigen-presenting cells, such as astrocytes that do not constitutively express MHCII [10–12]. This process was demonstrated in primary cultured astrocytes from rodents [13–16] and humans [17, 18]. MHCII expressed by astroglia appears to be involved in the pathophysiology of neuroinflammatory disorders, in particular in multiple sclerosis in humans and experimental autoimmune encephalomyelitis in animal models (EAE) [8, 9, 19–21]; however, the extent to which IFNγ-treated astrocytes can function as immunocompetent cells is still incompletely understood [10, 12]. Astrocyte-specific deficiency of the MHC class II transactivator, a master regulator of MHCII expression, does not affect induction of EAE [22]. However, as was shown recently, the expression of genes responsible for antigen presentation by MHCII is strongly induced in astrocytes in disease-affected brain regions in EAE [19]. Additionally, MHCII expression in the spinal cord correlates positively with IFNγ production and disease progression in EAE [21]. Thus, while astrocytes are capable of activating T cells during EAE, astrocytic MHCII molecules are not required for disease induction, but most likely play a complex (destructive) role in potentiation and exacerbation of ongoing disease [9, 10]. In particular, it is still unknown how MHCII molecules are relocated to the cell surface, where they perform their function. A recent study by Vardjan et al. demonstrated co-localization of immunolabeled MHCII with both LAMP1 and fluorescent dextrans (pulse loaded into cells for 16 h) at a qualitative level, indicating MHCII distribution within the endo-/lysosomal system of IFNγ-treated astrocytes [16]. However, the detailed subcellular localization and specific mechanism(s) by which MHCII molecules translocate to and are retained at the cell surface have never been studied.

We thus examined subcellular compartmentalization and surface exposure of MHCII in individual IFNγ-treated astrocytes using immunocytochemistry in conjunction with confocal and super-resolution structured illumination microscopy (SIM). Using high-resolution patch-clamp membrane capacitance monitoring, we measured, for the first time, how IFNγ treatment modifies interactions of single vesicles with the astrocyte plasmalemma. In IFNγ-treated astrocytes, MHCII predominately localizes to lysosomes that traffic toward the cell periphery and fuse with the plasmalemma to expose MHCII molecules at the surface. Concomitant suppression of endocytosis prolongs the presence of antigen at the cell surface. Thus, in aspects of vesicle traffic and exo-/endocytotic function pertinent to antigen processing and presentation, IFNγ-treated astrocytes display remarkable resemblance to specialized, professional antigen presenting immune cells.

## Materials and methods

### Culture of primary astrocytes

Primary astrocyte cultures were prepared from cerebral cortices of 2- to 3-day-old female Wistar rats as described previously [23]. Animal handling was in accordance with the International Guiding Principles for Biomedical Research Involving Animals developed by the Council for International Organizations of Medical Sciences and the Directive on Conditions for Issue of License for Animal Experiments for Scientific Research Purposes (Official Gazette of the Republic of Slovenia 40/85 and 22/87). Isolated cells were maintained in high-glucose Dulbecco’s modified Eagle’s medium (DMEM), supplemented with 10% fetal bovine serum, 1 mM sodium pyruvate, 2 mM l-glutamine, and 25 µg/ml penicillin/streptomycin in an atmosphere of 5% CO_2_/humidified air (95%) at 37 °C. Sub-confluent cultures were shaken at 225 rotations per minute overnight with a subsequent medium exchange; this was repeated three times. After astrocyte enrichment, cells were detached from the culture flask with 0.1% trypsin and 0.04% EDTA in Hank’s balanced salt solution, plated onto poly-l-lysine-coated glass coverslips, and bathed with the culture medium. Experiments were carried out 1–4 days after cell plating. IFNγ was added to the culture medium to reach a final concentration of 600 U/ml (Hycult Biotech, Uden, The Netherlands) for 48 h at 37 °C. All chemicals were purchased from Merck (Darmstadt, Germany) unless stated otherwise. All experiments were performed on primary astrocyte cultures unless stated otherwise.

### Solutions

The extracellular bath and pipette solution for electrophysiologic experiments consisted of 130 mM NaCl, 5 mM KCl, 2 mM CaCl_2_, 1 mM MgCl_2_, 10 mM d-glucose, and 10 mM HEPES/NaOH (pH 7.2). Solution osmolarity (300 ± 15 mOsm) was measured with a freezing-point osmometer (Osmomat 030, Gonotec, Berlin, Germany). ATP was added to the extracellular solution as a bolus to reach a final concentration of 100 µM.

### Plasmid and cell transfection

To visualize LAMP1-containing late endo-/lysosomes, we transfected astrocytes with the plasmid encoding LAMP1-EGFP (a gift from Dr. Magdalene So, Oregon Health Science University, Portland, OR, USA) using Lipofectamine LTX Reagent (Thermo Fisher Scientific). DNA (0.8 µg/µl) was mixed with 1 µl of PLUS Reagent, diluted in 50 µl of serum-free culture medium; 2 µl of Lipofectamine LTX Reagent was diluted in 50 µl of serum-free culture medium. Both solutions were mixed and incubated for 5 min at room temperature (RT). Astrocytes were washed and incubated in 900 µl of serum-free culture medium to which 100 µl of the lipofection mixture was added. Transfected astrocytes were incubated for 3 h at 37 °C in an atmosphere of 5% CO_2_/95% air; thereafter, 30 µl of Ultroser G (Pall, Port Washington, NY, USA) was added to the culture, and the culture was supplied with IFNγ (600 U/ml). The medium was exchanged for fresh culture medium containing IFNγ (600 U/ml) the next day. Transfected astrocytes were observed after 48 h.

### Immunocytochemistry and fluorescence co-localization analysis

We characterized the structure of MHCII-immunopositive vesicles in astrocytes fixed in formaldehyde (4%) by examining the fluorescence co-localization of immunolabeled MHCII and fluorescent LAMP1-EGFP, or immunolabeled Rab7, Rab4A, EEA1, and TPC1, all being markers of distinct endosomal compartments [24, 25]. Transfected cells were washed (3 min) with phosphate-buffered saline (PBS) and fixed in formaldehyde (4% in PBS) for 15 min, permeabilized with 0.1% Triton X-100 for 10 min and then washed four times with PBS, all at RT. The non-specific background staining was reduced by cell incubation in a blocking buffer with 10% (v/v) goat serum in 3% (w/v) bovine serum albumin (BSA) in PBS for 1 h at 37 °C. Cells were then washed with PBS once and incubated with primary antibodies diluted in 3% BSA in PBS overnight at 4 °C. The following primary antibodies were used: mouse monoclonal anti-MHCII (MRC-OX6 [26]; recognizes both non-loaded and peptide-loaded MHCII [27]; 1:100–1:250; ab23990, Abcam, Cambridge, UK), rabbit monoclonal anti-Rab7 (1:200; ab137029, Abcam), rabbit polyclonal anti-Rab4A (1:400; ab13252, Abcam), rabbit polyclonal anti-EEA1 (early endosomal antigen 1, 1:500; ab2900, Abcam), and rabbit polyclonal anti-TPC1 (two-pore channel 1, clone 3526#6C, 1:500; a gift from Prof. Dr. Norbert Klugbauer, Albert-Ludwigs-University, Freiburg, Germany). The next day, the cells were washed four times in PBS and stained with secondary anti-rabbit or anti-mouse antibodies conjugated to Alexa Fluor 546 or 488 (1:600; Thermo Fisher Scientific), respectively, for 45 min at 37 °C, and then washed four times in PBS. Finally, coverslips were mounted onto glass slides using SlowFade Gold antifade mountant with or without DAPI (Thermo Fisher Scientific).

In some experiments, IFNγ-treated cells were incubated in culture medium containing 0.5‰ DMSO (v/v; vehicle) or 200 µM GPN (Santa Cruz Biotechnology, Dallas, TX, USA) for 30 min at 37 °C immediately before MHCII immunolabeling.

Immunocytochemical labeling of live cells was performed as described previously [28]. Cells were first washed once with ice-cold 3% BSA in PBS and incubated with anti-MHCII antibodies diluted in 3% BSA in PBS for 30 min on ice. Subsequently, cells were washed three times with ice-cold 3% BSA in PBS and incubated with corresponding secondary antibodies for 30 min on ice. Finally, cell-loaded coverslips were washed three times with ice-cold 3% BSA in PBS and once with the extracellular solution, and transferred to the recording chamber. For concurrent labeling of cell surface in live, immunolabeled astrocytes, cells were bathed with extracellular solution containing 4 µM plasmalemmal marker FM4-64 (Thermo Fisher Scientific).

Immunofluorescently labeled cells were observed with a confocal microscope (LSM 780, Zeiss) with a plan apochromatic oil-immersion objective 63 × /NA 1.4. Confocal images (single planes or z stacked) were obtained with a 488-nm argon laser and 561-nm diode-pumped solid-state laser excitation, and the fluorescence emission was band-pass filtered at 500–550 nm (or 495–530, EGFP) and 565–615 nm, respectively.

To quantify the co-localization of red-emitting (immunofluorescent MHCII) and green-emitting fluorophores (LAMP1-EGFP or immunofluorescent Rab7, Rab4A, EEA1, and TPC1), tiff files were exported and analyzed with ColocAna (Celica Biomedical, Ljubljana, Slovenia), which enables automated high-throughput co-localization analysis of fluorescent markers in a large number of images [29]. Briefly, the program counted all green, red, and co-localized (green and red) pixels in each image. The fluorescence co-localization (%) was determined with reference to green pixels identifying specific endosomal or lysosomal compartments. The threshold for the co-localized pixel count was set to 20% of the maximal fluorescence to reduce apparent fluorescence overlap originating from closely positioned fluorescent structures. In live cell immunolabeling, the fluorescence co-localization between red-emitting FM4-64 and green-emitting immunofluorescent MHCII was quantified.

### LysoTracker labeling

Acidic late endo-/lysosomes were labeled by incubating cells in culture medium containing 200 nM LysoTracker red DND-99 (Thermo Fisher Scientific) for 5 min at 37 °C. LysoTracker-labeled cells were washed once with extracellular solution, mounted onto the recording chamber, supplied with bath solution, and exposed to either 0.5‰ DMSO or 200 µM GPN during observation on a confocal microscope (LSM 780, Zeiss).

### CT-B conjugate labeling

Cholera toxin B subunit Alexa Fluor 488 conjugate (CT-B; Thermo Fisher Scientific) labeling was performed as described previously [28]. Briefly, non-treated and IFNγ-treated astrocytes were washed three times in 3% BSA in PBS and once with DMEM and incubated in culture medium with 1 µg/ml of CT-B for 10 min at 4 °C. The astrocytes were then washed twice with ice-cold PBS and incubated with anti-CT-B antibodies (1:200) diluted in culture medium for 15 min at 4 °C. Labeled astrocytes were washed twice with ice-cold PBS, and MHCII was subsequently immunolabeled as described above.

### Structured illumination microscopy and image analysis

Immunofluorescent and CT-B labeled astrocytes were imaged with an oil-immersion plan apochromatic differential interference contrast objective (63 × /NA 1.4) using a super-resolution SIM microscope (ELYRA PS.1, Zeiss). Fluorescent images were acquired with an EMCCD camera (andor iXon 885, Andor Technology, Belfast, UK) using five grating frequencies for SIM. Alexa Fluor 488 and Alexa Fluor 546 were excited with the 488 nm and 561 nm diode-pumped solid-state laser lines, and emission fluorescence was filtered with 495−575 nm and 570−650 nm band-pass filters, respectively. To assess the subcellular localization of MHCII-positive vesicles, separation of the periplasmalemmal space and the cell interior was performed with a custom-written MATLAB program (MathWorks, Natick, MA, USA) that enabled temporary adjustments of the image brightness. The CT-B-labeled plasmalemma was manually outlined along the outer rim after which the program eroded a 20.5-pixel (810-nm) wide band, with 22% of the width residing outside and 78% residing inside the labeled perimeter. The circumference of the outlined cell was calculated, and two tiff images were generated, one depicting the cell interior and the other depicting the periplasmalemmal space.

To estimate the apparent vesicle size (vesicle image area), confocal and SIM images were analyzed with ImageJ software (NIH, Bethesda, MD, USA; https://rsbweb.nih.gov/ij/). The minimum fluorescent spot taken to identify an individual vesicle in a confocal image was three adjacent pixels (0.176 × 0.176 μm), and the minimum surface area covered by a spot was 0.093 µm^2^. The number of internalized vesicles per cell image was analyzed by considering that a minimum MHCII-positive vesicle consisted of three adjacent pixels; thus, a broad span of vesicles with different sizes was covered by the analysis. In super-resolution SIM micrographs, the minimum spot taken to identify an individual vesicle consisted of five adjacent pixels (0.040 × 0.040 μm), and the minimum surface area (S) covered by the particle was 0.008 µm^2^.

### Preparation and treatment of rat organotypic brain slices

Organotypic brain slice cultures were prepared from 9- to 10-week-old male Wistar rats by the membrane interface method using a protocol modified from De Simoni and Yu [30]. Briefly, animals were killed by exposure to 100% CO_2_ for ~ 5 min and immediately decapitated. The brains were carefully removed and immersed in ice-cold slicing medium containing Hank’s balanced salt solution and an antibiotic–antimycotic mixture (100 units/ml penicillin, 100 µg/ml streptomycin and 0.25 µg/ml amphotericin B; Thermo Fisher Scientific, Waltham, MA, USA). The cerebellum and frontal section (approximately one-third) of the brain were cut with a scalpel blade, and the frontal aspect of the brain was glued onto the cutting table of a vibratome (VT1000 S, Leica Biosystems, Wetzlar, Germany). Coronal slices with a thickness of 160 µm were cut and submerged in ice-cold slicing medium throughout the sectioning process. These slices were transferred for cultivation to sections of LCR (hydrophilic polytetrafluoroethylene) membrane (FHLC02500, Merck) on the bottom of membrane inserts with a pore size of 0.45 µm. Membrane inserts were prepared 1 day before culturing by placing them in 6-well culture plates containing slice culture medium consisting of 50% minimum essential medium (Thermo Fisher Scientific), 25% Eagle’s balanced salt solution, 23.75% horse serum (Thermo Fisher Scientific), 1 mM l-glutamine, 21 mM d-glucose and antibiotic–antimycotic mixture (Thermo Fisher Scientific). Two sections of sterile LCR membrane were placed into each membrane insert. Slices were maintained at 37 °C in an atmosphere of 5% CO_2_/95% air. The culture medium was changed on the first day after plating and then on every second day. For IFNγ astrocyte treatment, IFNγ was added to the slice culture medium to reach a final concentration of 600 U/ml (Hycult Biotech) for 48 h. All chemicals were purchased from Merck unless stated otherwise.

### Immunohistochemistry of organotypic brain slices and image analysis

Organotypic brain slices cultured on top of sections of LCR membrane were fixed in formaldehyde (4% in PBS) for 4 h at RT, washed four times (15 min each) with PBS, blocked, and permeabilized with 20% BSA and 0.5% Triton X-100 in PBS overnight at 4 °C. The next day, slices were incubated with primary antibodies (rabbit monoclonal anti-GFAP, 1:200, ab33922, Abcam; and mouse monoclonal anti-MHCII, 1:100, ab23990, Abcam) diluted in 2% BSA and 0.25% Triton X-100 in PBS for 24 h at 4 °C. Labeled slices were washed four times (15 min each) in 2% BSA in PBS and stained with secondary anti-rabbit and anti-mouse antibodies conjugated to Alexa Fluor 546 or 488 (1:600; Thermo Fisher Scientific), respectively, for 2 h at 37 °C, and then washed four times (15 min each) in PBS. Finally, slices were supplied with 30 µM DAPI (Thermo Fisher Scientific) in PBS for 5 min at RT, washed three times with PBS (5 min each), mounted onto glass slides using VECTASHIELD Antifade Mounting Medium (Maravai LifeSciences, San Diego, CA, USA), and sealed with translucent nail polisher.

Immunofluorescent cells were observed with a confocal microscope (LSM 780, Zeiss) using a plan apochromatic oil-immersion objective 63 × /NA 1.4. Multichannel z-stacked images were acquired by successive cell illumination with 405-nm diode-pumped solid-state laser, 488-nm argon laser, and 561-nm diode-pumped solid-state laser, and the fluorescence emission was band-pass filtered at 440–480 nm (DAPI) at 500–550 nm (Alexa 488) and 565–615 nm (Alexa 546), respectively.

To quantify the extent by which exposure to IFNγ alters the expression of MHCII in GFAP-immunopositive hippocampal astrocytes in organotypic brain slices, the maximum intensity orthogonal projections were generated from z-stacked confocal images and exported as tiff files to ImageJ for analysis. To estimate the territory (area) of individual GFAP-positive astrocytes, GFAP fluorescence was first thresholded using the mean thresholding method to obtain the binarized mask images. Holes within the mask images were filled by the Fill Holes image processing tool, and the area of filled mask was delimited and measured. The area of MHCII-positive vesicles within the territory of a given GFAP-positive astrocyte was measured as stated in the previous section. To calculate the relative cell surface covered by MHCII-positive vesicles in controls and IFNγ-treated astrocytes, the cumulative MHCII vesicle area was divided by the corresponding GFAP cell area.

### Electrophysiology

Astrocyte-loaded coverslips were placed into the recording chamber, supplied with extracellular solution, and mounted on an inverted microscope (Zeiss Axio Observer, Zeiss). Cell-attached membrane capacitance recordings were performed with a dual-phase lock-in patch-clamp amplifier (SWAM IIC, Celica Biomedical) at RT with fire-polished, standard-walled borosilicate glass pipettes (30-0058, Harvard Apparatus, Holliston, MA, USA) of 2–6 MΩ, heavily coated with the silicon resin Sylgard 184 (Dow Corning, Midland, MI, USA). The exo-/endocytotic activity of individual astrocytes was recorded by the compensated cell-attached patch-clamp technique, enabling measurements of discrete stepwise increases and decreases in membrane capacitance (*C*_m_) [31, 32]. Cell membrane was voltage clamped at a holding potential of 0 mV to which a sine wave voltage (111 mV root mean square) was applied at a frequency (*f*) of 6400 Hz. The phase angle of the lock-in amplifier was adjusted to nullify changes in the real (Re) part of the admittance signal in response to 10 fF calibration steps in the imaginary (Im) part of the signal. The 10-fF calibration pulses were triggered manually with a built-in calibration circuit every ~ 15 s to ensure correct phase angle settings [33]. Re and Im signals were low-pass filtered at 100 Hz, whereas membrane current was low-pass filtered at 10 Hz (4-pole Bessel filter, − 3 dB); all were digitized at an acquisition rate of 200 Hz.

For exo-/endocytotic events observed in Im, displaying or not a projection to the Re, the vesicle capacitance (*C*_v_) was calculated from *C*_v_ = [(Re^2^ + Im^2^)/Im]/*ω*, where *ω* denotes angular frequency (*ω* = 2*πf*). If no projection is displayed to the Re trace, ∆Im is directly proportional to ∆*C*_m_. The projection to Re reflects the formation of a narrow fusion pore, which introduces a new highly resistive element to the electrical circuit [34–36]. As *C*_m_ is proportional to the plasma membrane area, the vesicle surface area and thus its diameter (*d*) can be determined from *C*_v_ according to the equation *C*_v_ = C_spec_*πd*^2^, where *C*_spec_ denotes the specific membrane capacitance. Vesicle diameter was calculated by assuming spherical geometry and by using a *C*_spec_ of 10 fF/µm^2^ [37]. The presence of Re projections enabled us to calculate the conductance of narrow fusion pores: *G*_p_ = (Re^2^ + Im^2^)/Re, and subsequently to estimate the fusion pore diameter (2*r*) using the equation *G*_p_ = (*πr*^2^)/(*ρλ*), where *ρ* is saline resistivity (100 Ω cm) and *λ* is the estimated fusion pore length (15 nm) [38].

Events in Im were manually selected by the cursor option in CellAn (Celica Biomedical) written for MATLAB. An event was considered detectable if the signal-to-noise ratio was at least 3:1, and the event did not exhibit projection to the current trace. An event was considered reversible (reversible exo-/endocytosis) if a step in Im was followed by a subsequent step of the same amplitude and opposite direction within 15 s, and irreversible (full exo-/endocytosis) in the absence of a reciprocal step. Time-dependent changes in Im were recorded in non-stimulated and ATP-stimulated (100 μΜ) cells that were either treated or not with IFNγ for 48 h. ATP was added to the recording chamber as a bolus to reach a final concentration of 100 µM.

### Assessment of dextran uptake

To assess how IFNγ treatment affects bulk fluid-phase endocytosis, non-treated control and IFNγ-treated astrocytes were incubated in culture medium containing 10 µM of 10 kDa dextran Alexa Fluor 488 conjugate (Dex488; Thermo Fisher Scientific) and 600 U/ml IFNγ (only with IFNγ-treated astrocytes) for 3 h at 37 °C. After incubation, Dex488-labeled cells were washed two times with extracellular solution, mounted onto the recording chamber, supplied with bath solution and observed by a confocal microscope (LSM 780, Zeiss).

### Statistical analysis

The relative proportion of MHCII-positive cell area, number and surface area of immunolabeled MHCII vesicles, single-vesicle capacitance, apparent pore dwell time and fusion pore conductance, and frequency of reversible and full exo-/endocytotic events are expressed as means ± SEM (standard error of the mean). Statistical significance was determined with the Mann–Whitney *U* test or ANOVA on ranks followed by Dunn’s test using SigmaPlot 11.0 (Systat Software, San Jose, CA, USA).

## Results

### MHCII is localized in late endosomes and lysosomes of IFNγ-treated astrocytes

**Fig. 1:**
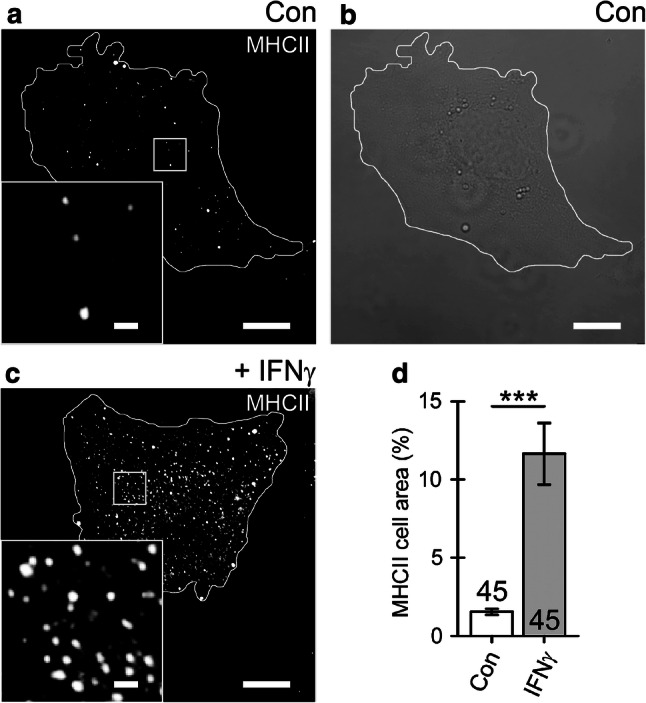
Cell treatment with IFNγ enhances the expression of MHCII that localize to vesicle-like structures in cultured rat astrocytes. **a** Confocal image of control (Con) astrocyte immunolabeled by anti-MHCII and secondary Alexa-546-conjugated antibody. **b** Differential interference contrast image of the same cell as in (**a**). **c** Confocal image of an astrocyte treated with IFNγ for 48 h. The white curve outlines the cell perimeter (**a**–**c**). Note numerous MHCII-positive vesicles in an IFNγ-treated astrocyte observed as bright fluorescent puncta. Insets display a magnified view of the MHCII-positive vesicles in control and IFNγ-treated cells. Scale bars: 10 μm (large images **a**–**c**) and 1 μm (insets **a**, **c**). **d** The relative proportion of MHCII-positive cell area (%; surface area of MHCII-positive pixels with fluorescence above 20% of maximal fluorescence) normalized to cell image area (surface area of all pixels delimited by the cell perimeter). The MHCII-positive cell area is substantially higher in IFNγ-treated astrocytes. The numbers at the bottom of the bars indicate the number of cell images analyzed. ***P < 0.001 versus control (Mann–Whitney *U* test)

To study the subcellular distribution of MHCII in rat astrocytes, cells were maintained in purified culture and treated with IFNγ for 48 h to induce expression of MHCII [13–16]. This resulted in the appearance of numerous MHCII-positive immunofluorescent puncta distributed throughout the cytoplasm of IFNγ-treated cells, whereas in non-treated controls only scarce fluorescent puncta were observed (Fig. 1a–c). The relative cell area covered by MHCII-positive immunofluorescence was ~ 8 times larger in IFNγ-treated cells than in non-treated controls (Fig. 1d). Increased expression of MHCII-positive fluorescence was also observed in GFAP-positive hippocampal astrocytes in organotypic brain slices exposed to IFNγ for 48 h but not in GFAP-positive astrocytes in control, non-treated slices (Online Resource 1, Fig. S1). The relative MHCII-positive cell area (normalized to the GFAP cell area) was ~ 21 times larger in IFNγ-treated astrocytes compared with non-treated controls (Online Resource 1, Fig. S1i). Apparent expression of GFAP also increased in IFNγ-treated astrocytes when compared with non-treated controls (Online Resource 1, Fig. S1a,b).

**Fig. 2:**
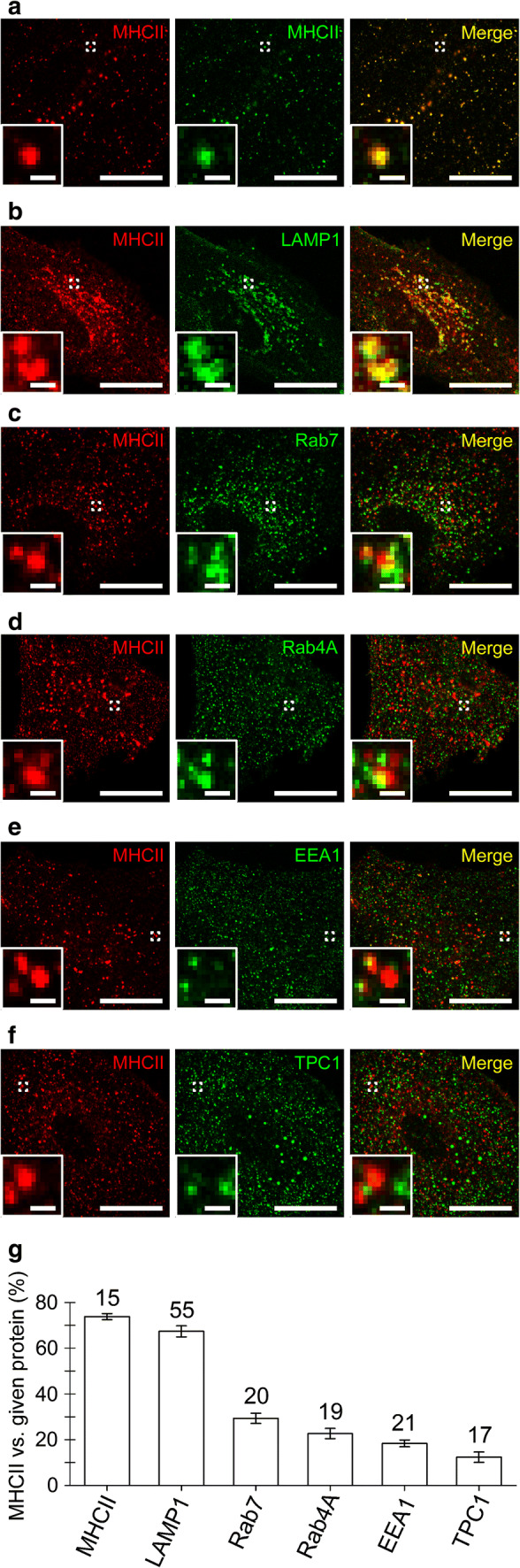
MHCII localize predominantly to lysosomes, but not early and recycling endosomes. **a**–**f** Confocal micrographs of fixed double-fluorescent IFNγ-treated astrocytes displaying immunolabeled MHCII (red, left) and compartments labeled by the primary antibody against MHCII (**a**), LAMP1-EGFP (**b**), primary antibodies against Rab7 (**c**), Rab4A (**d**), EEA1 (**e**), TPC1 (**f**), and the corresponding fluorescent secondary antibodies (green, middle). The merged images display co-localized pixels (yellow, right); insets display a magnified view of the selected vesicles (white open frame). Scale bars: 20 μm (large images) and 1 μm (insets) (**a**–**f**). (**g)** Graph displaying quantitative co-localization (%, mean ± SEM) of anti-MHCII fluorescence versus anti-MHCII, LAMP1-EGFP, anti-Rab7, anti-Rab4A, anti-EEA1, and anti-TPC1 fluorescence. The numbers above the bars indicate the number of cell images analyzed

Immunolabeled MHCII predominantly co-localized with LAMP1-EGFP, a protein characteristic of lysosomes (Fig. 2b, g), modestly with Rab7 (Fig. 2c, g), a protein characteristic of late endosomes and multivesicular bodies as well as of autophagosomes and lysosomes, and scarcely with Rab4A, EEA1, and TPC1 (Fig. 2d–g), proteins characteristic of early and recycling endosomes [24, 25]. Quantitative fluorescence co-localization of immunolabeled MHCII was as follows: 72 ± 2% (mean ± SEM, *n* = 15) with anti-MHCII, 67% ± 2% (*n* = 55) with LAMP1-EGFP, 29% ± 2% (*n* = 20) with anti-Rab7, 23% ± 2% (*n* = 19) with anti-Rab4A, 18% ± 1% (*n* = 21) with anti-EEA1, and 12% ± 2% (*n* = 17) with anti-TPC1 (Fig. 2g). These data indicate that MHCII-immunopositive organelles are lysosomes, which undergo Ca^2+^-dependent exocytosis in astrocytes [39–44].

**Fig. 3:**
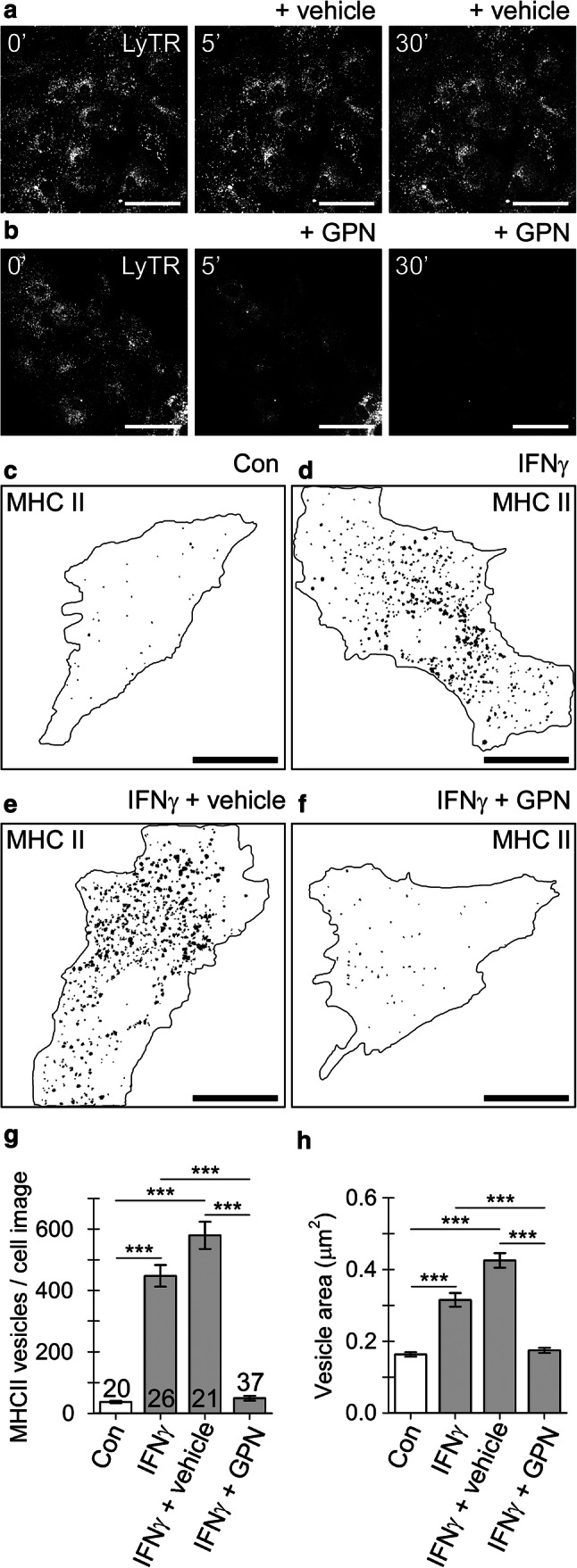
IFNγ-induced localization of MHC II into astrocyte lysosomes is disrupted by a lysosomolytic agent glycyl-l-phenylalanine-β-naphthylamide (GPN). **a** and **b** Confocal images of live astrocytes labeled with LysoTracker red DND-99 (LyTR) before (left), 5 min (middle), and 30 min (right) after addition of vehicle (0.5‰ v/v DMSO) (**a**) or 200 µM GPN (**b**). Scale bar: 50 µm. **c**–**f** Binarized images of control astrocyte (Con), IFNγ-treated astrocyte (IFNγ), IFNγ-treated astrocyte exposed to vehicle (IFNγ + vehicle), and IFNγ-treated astrocyte exposed to 200 µM GPN for 30 min (IFNγ + GPN). Numerous black puncta in the mask images depict individual MHCII-positive vesicles composed of ≥ 3 adjacent pixels with mean fluorescence intensity > 20% of maximal fluorescence. Note the diminished number of MHCII-positive vesicles in the IFNγ-treated astrocyte exposed to GPN. The black curve outlines the cell perimeter (**c**–**f**). Scale bar: 20 µm. **g**–**h** Number (mean ± SEM) of MHCII-positive vesicles (**g**) and the mean vesicle surface area per imaged cell (**h**) in non-treated controls (Con), IFNγ-treated astrocytes (IFNγ), IFNγ-treated astrocytes exposed to vehicle (IFNγ + vehicle), and IFNγ-treated astrocytes exposed to 200 µM GPN for 30 min (IFNγ + GPN). Numbers at the base of the bars indicate the number of cells analyzed. ***P < 0.001 versus respective comparison (ANOVA on ranks followed by Dunn’s test)

To further confirm the localization of MHCII into late endo-/lysosomes, we applied a lysosomotropic agent glycyl-l-phenylalanine 2-naphthylamide (GPN), which disrupts lysosomes to astrocytes in cell cultures [45, 46]. When cells were labeled with LysoTracker red (LyTR), a fluorescent weak base accumulated inside acidified organelles in the perinuclear cell region (Fig. 3a, b). After the cells were treated with 200 µM GPN for 30 min, a profound reduction in LyTR fluorescence was observed (Fig. 3b), which was not the case when cells were treated with vehicle (0.5‰ DMSO), consistent with the labeling of structurally intact lysosomes (Fig. 3a). Analysis of binarized, confocal mask images in non-treated controls and IFNγ-treated cells (Fig. 3c–f) revealed that cell treatment with GPN substantially affected the number and size of MHCII-positive vesicles. In non-treated controls, a low number of MHCII-positive vesicles was observed (37 ± 4 vesicles per cell image), whereas in IFNγ-treated cells, a high number of vesicles was observed (580 ± 44 and 447 ± 35 vesicles for IFNγ and IFNγ + vehicle, respectively). Exposure of IFNγ-treated cells to 200 µM GPN reduced the number of MHCII-positive vesicles to 49 ± 8 vesicles per cell image (Fig. 3g). Exposure to GPN also diminished the apparent size of MHCII-positive vesicles in IFNγ-treated cells, where vesicles with much larger areas were typically observed (0.43 ± 0.02 µm^2^ and 0.32 ± 0.02 µm^2^ for IFNγ and IFNγ + vehicle, respectively) than in non-treated controls and in IFNγ-treated cells exposed to GPN (0.16 ± 0.01 µm^2^ and 0.17 ± 0.01 µm^2^, respectively; Fig. 3h). Collectively, these data also indicate that astrocytic MHCII predominantly resides in late endo-/lysosomes.

### MHCII-positive organelles constitute a subpopulation of larger vesicles positioned in the vicinity of the astrocyte plasmalemma

**Fig. 4:**
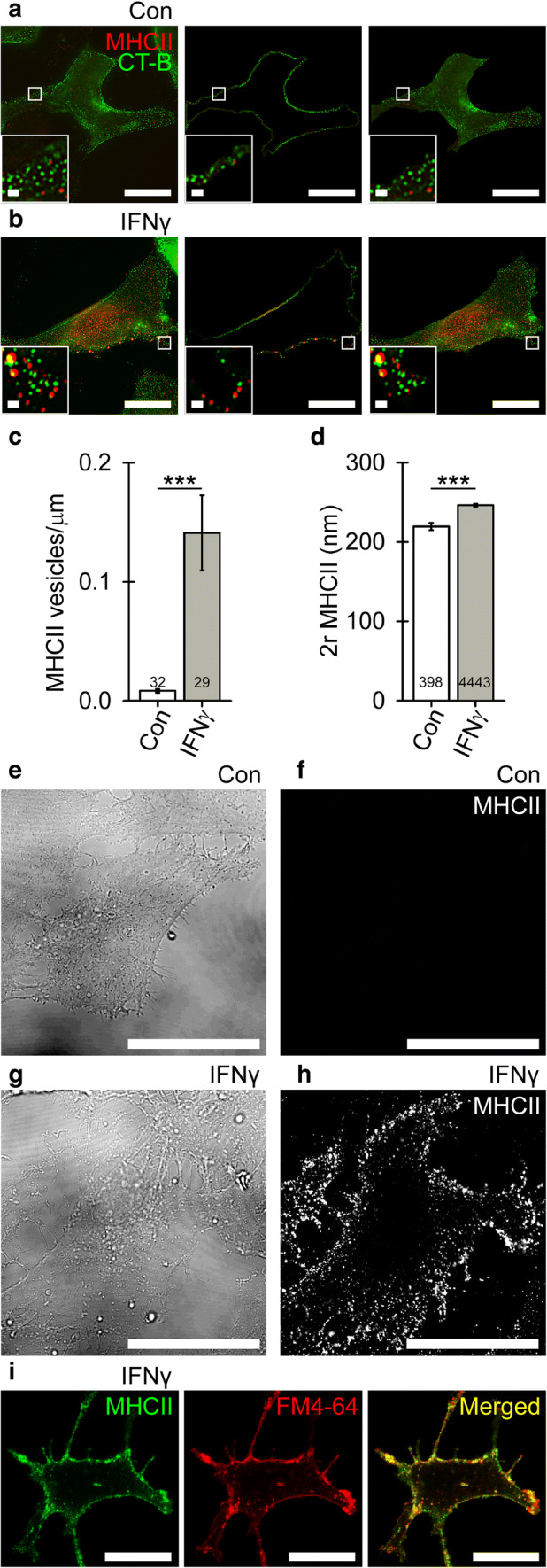
IFNγ treatment induces the formation of large MHCII-positive vesicles in the vicinity of the plasmalemma (**a** and **b**) Super-resolution SIM images of double-fluorescent astrocytes labeled with cholera toxin subunit B Alexa 488 conjugate (CT-B; green) and anti-MHCII tagged with fluorescent secondary antibody (MHCII; red) in controls (**a**) and IFNγ-treated cells (**b**). The number of MHCII-positive vesicles was quantified in a region-dependent manner in images of the cell periphery (middle) and cell interior (right); for details see “Materials and methods”. Scale bars: 20 µm (large image) and 1 μm (insets). **c **Number of MHCII-positive vesicles in the cell periphery per micrometer of plasmalemma in control and IFNγ-treated astrocytes (mean ± SEM). **d** Diameter (2*r*) of MHCII vesicles in control and IFNγ-treated astrocytes. Numbers at the bottom of the bars indicate the number of cells (**c**) or vesicles (**d**) analyzed. ***P < 0.001 (Mann–Whitney *U* test). **e** and **g** differential interference contrast images of live control (Con; **e**) and IFNγ-treated astrocytes (IFNγ, **g**), and the corresponding confocal images displaying MHCII-positive immunofluorescence at the cellular surface (**f** and **h**). Note abundant MHCII-positive immunofluorescence at the surface of IFNγ-treated (**h**) but not control cells (**f**). Scale bar: 20 µm. (i) Confocal micrographs of double-fluorescent, non-permeabilized, IFNγ-treated astrocytes labeled with anti-MHCII tagged with fluorescent secondary antibody (MHCII, green, left) and styryl dye FM4-64 (red, middle). The merged image displays co-localized pixels (yellow, right). Scale bar: 20 µm

Next, we asked whether MHCII-positive late endo-/lysosomes are delivered toward the astrocyte plasmalemma, where they may undergo exo-/endocytosis [39–44]. Non-treated controls and IFNγ-treated cells were double labeled by CT-B, which stains plasmalemmal domains enriched in ganglioside monosialic acid (GM1) lipid rafts [47], and by anti-MHCII, which stains individual MHCII-positive vesicles. Using super-resolution SIM microscopy, we assessed the subcellular localization of MHCII-positive vesicles and estimated their diameter at high lateral resolution [48, 49]. First, we counted vesicles in images of the cell periphery, defined as an 810-nm wide periplasmalemmal band, and the cell interior, and normalized their number per plasmalemma length (µm^−1^). In non-treated controls, only a few vesicles were observed at the cell periphery, whereas in IFNγ-treated cells vesicles were much more abundant (0.01 ± 0.00 vesicles/µm versus 0.14 ± 0.03 vesicles/µm, respectively; Fig. 4c). The diameter of MHCII-positive vesicles was 216 ± 12 nm in controls versus 230 ± 4 nm in IFNγ-treated cells (Fig. 4d). These data demonstrate that cell treatment with IFNγ favored the formation of large MHCII-positive vesicles that were concentrated close to the plasmalemma. To confirm MHCII incorporation into the plasmalemma of IFNγ-treated cells, we applied anti-MHCII and the corresponding fluorescent secondary antibodies to non-permeabilized control astrocytes (Fig. 4e, f) and IFNγ-treated astrocytes (Fig. 4g, h) to label the cell surface exclusively. In control cells, no MHCII-positive structures were observed (Fig. 4f), whereas in IFNγ-treated cells, they were numerous and extended over large stretches of the cell surface (Fig. 4h). Additionally, in these experiments immunofluorescent MHCII strongly co-localized with the plasmalemmal marker FM4-64, the degree of co-localization vs. MHCII-positive immunofluorescence was 83 ± 1% (Fig. 4i).

### Astrocyte treatment with IFNγ promotes reversible exocytosis of larger vesicles and inhibits endocytosis

**Fig. 5:**
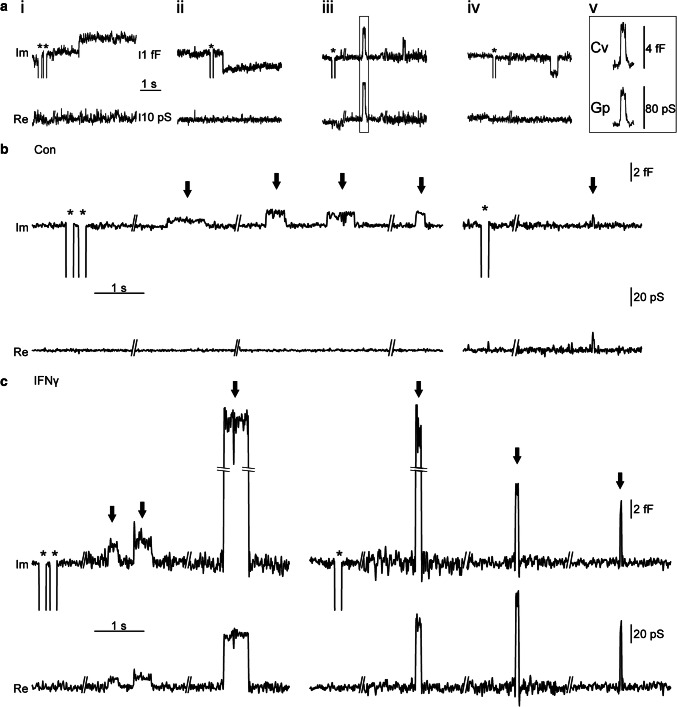
Types of vesicle–plasmalemma interactions measured by high-resolution patch-clamp membrane capacitance measurements in cultured rat astrocytes. **a** Representative examples of upward and downward steps in the imaginary (Im) and real (Re) component of the admittance signal represent elementary events of exo- and endocytosis: (i) full exocytosis (ii) full endocytosis (iii) reversible exocytosis (iv) reversible endocytosis; (v) an event of reversible exocytosis that exhibited projection to the Re trace, indicating the formation of a highly resistive narrow fusion pore enabling measurement of fusion pore conductance (*G*_p_). Asterisks denote calibration pulses. For details, see “Materials and methods”. **b** and **c** Individual events of reversible exocytosis in control (**b**) and IFNγ-treated astrocytes (**c**) marked by the arrows in Im component of the admittance trace signal

To examine the nature of vesicle interactions with the plasmalemma in IFNγ-treated astrocytes, we conducted high-resolution cell-attached membrane capacitance (*C*_m_) measurements to detect single-vesicle exo-/endocytotic events and to determine the dynamic properties of vesicle fusion pores [31, 33]. Over a total recording time of 26.8 h and an average time of 17.7 ± 0.4 min per recording, we examined 91 astrocytes that exhibited at least one discrete on and/or off step, representing unitary exocytotic and endocytotic events, respectively. As reported previously [33, 35, 50, 51], we observed distinct types of exo-/endocytotic events in both control and IFNγ-treated astrocytes (Fig. 5a–c). Full exo-/endocytotic events consisted of single on or off steps, indicating full fusion or full fission of the vesicle, respectively (Fig. 5ai, ii). Reversible exo-/endocytotic events represent fusion pore opening quickly followed by its closure, consist with an on/off step followed by an opposite step of the same amplitude within 15 s (Fig. 5aiii, iv). In sporadic bursting events [50, 51], we measured the average amplitude and dwell time of the first and last flicker in the burst and pooled these data together with the data for reversible exo-/endocytotic events. The absolute number and the relative proportion of the type of vesicle interactions with the plasmalemma are presented in Table 1.

**Table 1:** Absolute number and relative frequency (%) of elementary exo-/endocytotic events in the studied groups

	Con (47 cells)		Con + ATP (28 cells)		IFNγ (44 cells)		IFNγ + ATP (29 cells)	
	Count	%	Count	%	Count	%	Count	%
Full exocytosis	82	9	54	13	51	9	46	13
Full endocytosis	406	47	103	25	238	41	81	24
Reversible exocytosis	298	34	168	41	206	36	160	47
Reversible endocytosis	84	10	88	21	82	14	56	16
Total	870		413		577		343	

*Con* controls, *Con + ATP* non-treated controls after ATP stimulation,* IFNγ* IFNγ-treated astrocytes, *IFNγ + ATP* IFNγ-treated astrocytes after ATP stimulation

**Fig. 6:**
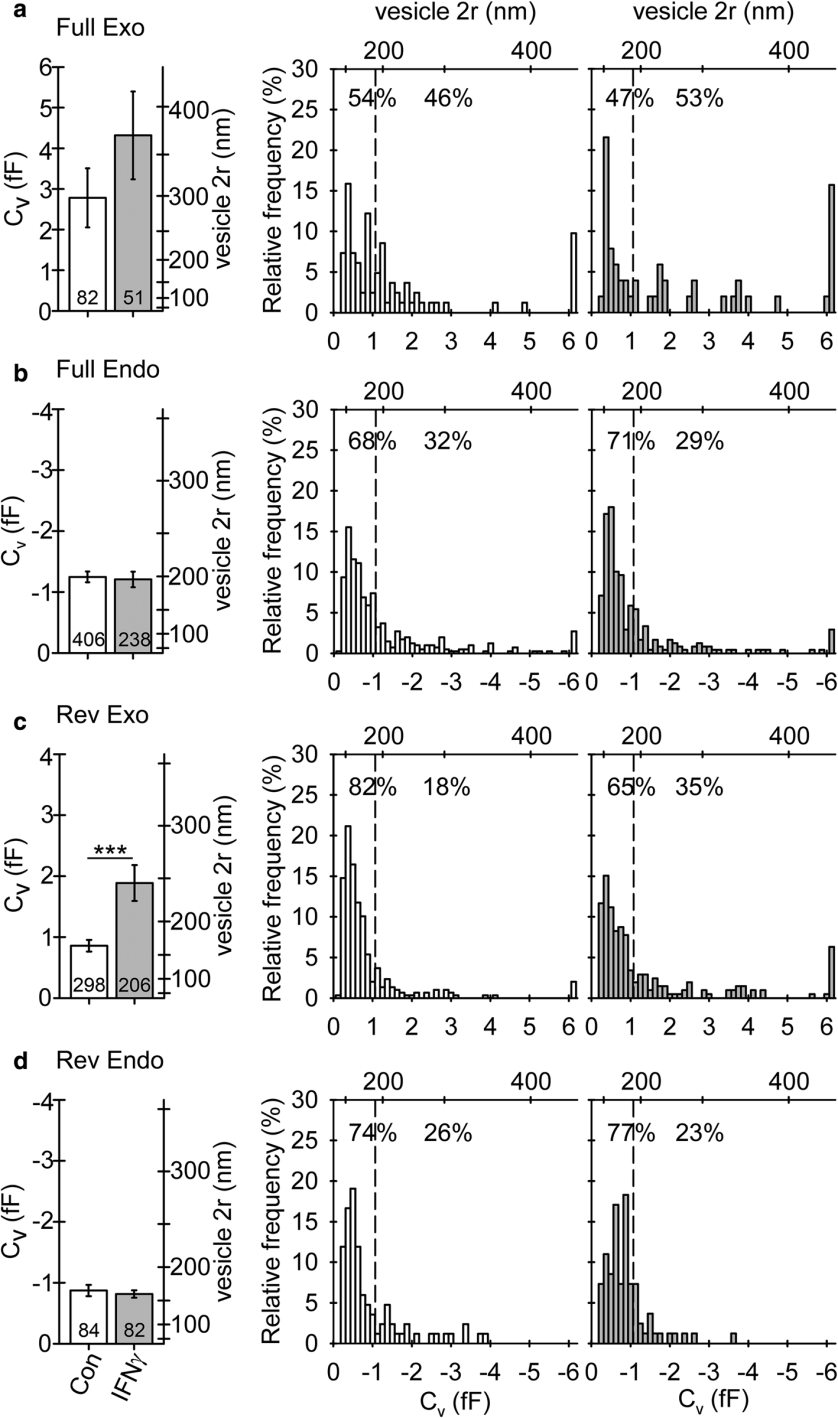
Astrocyte treatment by IFNγ favors reversible fusion of exocytotic vesicles with larger diameter (a–d) Plots depicting vesicle diameter (2*r*; mean ± SEM, left) and relative frequency distribution of vesicle capacitance (*C*_v_, bottom right) and vesicle diameter (2*r*, top right) in vesicles undergoing full exocytosis (**a**), full endocytosis (**b**), reversible exocytosis (**c**), and reversible endocytosis (**d**) in controls and IFNγ-treated astrocytes. Numbers at the bottom of the bars indicate the number of vesicles analyzed. In IFNγ-treated astrocytes, note an increase in vesicle diameter in exocytotic vesicles undergoing transient exocytosis. ***P < 0.001 (Mann–Whitney *U* test)

**Fig. 7:**
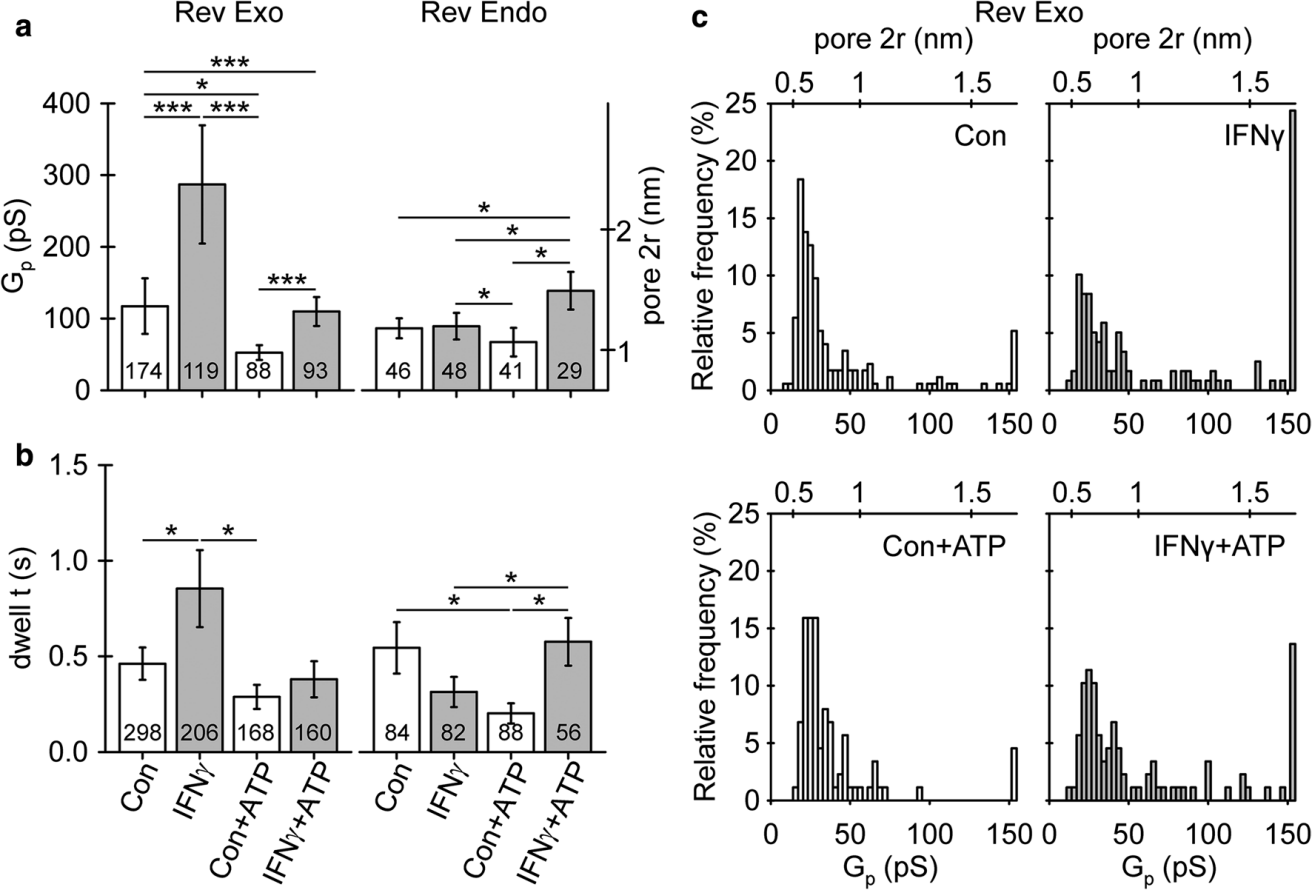
Astrocyte treatment with IFNγ alters fusion pore geometry and pore kinetics in reversible exocytosis. **a** Fusion pore conductance (*G*_p_) and diameter (2*r*) of reversible exo- (Rev Exo) and endocytotic vesicles (Rev Endo) establishing a narrow fusion pore at rest and after ATP stimulation of controls and IFNγ-treated astrocytes. **b** Fusion pore dwell time (dwell t) measured in reversible exo- and endocytotic vesicles at rest and after ATP stimulation in controls and IFNγ-treated astrocytes. Numbers at the bottom of the bars denote the number of vesicles (**a**), and exo- and endocytotic events examined (**b**). Note an increase in fusion pore diameter and open pore dwell time in vesicles undergoing transient exocytosis at rest and after ATP stimulation in IFNγ-treated astrocytes. *P < 0.05, **P < 0.01, ***P < 0.001 (Mann–Whitney *U* test). **c** Plots depicting the relative frequency distribution of fusion pore conductance (*G*_p_) and pore diameter (pore 2*r*) in reversible exocytotic vesicles establishing a narrow fusion pore at rest and after ATP stimulation in controls and IFNγ-treated astrocytes

The diameter (for calculations, see “Materials and methods”) of vesicles undergoing reversible exocytosis was significantly larger in IFNγ-treated versus non-treated astrocytes (194 ± 10 nm and 144 ± 5 nm, respectively; Fig. 6c). The diameter of vesicles undergoing full exocytosis, as well as reversible and full endocytosis, was similar in non-treated and in IFNγ-treated cells (Fig. 6a, b, d). The proportion of vesicles that entered reversible exo-/endocytosis and established a narrow fusion pore, as revealed by the projection of Im to Re (for details, see Materials and methods section), was similar in non-treated and in IFNγ-treated cells; 51 ± 5% versus 47 ± 6%, *p* = 0.61, and 51 ± 7% versus 52 ± 7%, *p* = 0.95, for reversible exocytosis and endocytosis, respectively (Table 2). In exocytotic vesicles, conductance of the narrow fusion pore and the open fusion pore dwell time was significantly larger in cells treated by IFNγ compared with non-treated controls: 287 ± 82 (*n *= 119) versus 117 ± 39 pS (*n* = 174, Fig. 7a) and 0.9 ± 0.2 s (*n* = 206) versus 0.5 ± 0.1 s (*n* = 298), respectively (Fig. 7b). We next examined the relationship between the size of vesicles and narrow fusion pores by calculating the correlation between vesicle capacitance (*C*_v_) and fusion pore conductance (*G*_p_). As observed previously [52], vesicle capacitance was positively correlated with fusion pore conductance; the apparent Pearson correlation coefficient (*r*) was 0.94 (*p* < 0.001) and 0.72 (*p* < 0.001) for control and IFNγ-treated astrocytes, respectively. We also observed a weak positive correlation between the vesicle capacitance and the open pore dwell time; the apparent Pearson correlation coefficient (*r*) was 0.17 (*p* < 0.05) and 0.22 (*p* < 0.05), respectively.

**Table 2:** Cumulative absolute number (count) and relative frequency per cell (%, mean ± SEM) of reversible exo-/endocytotic events exhibiting projections to the Re signal (w proj.), representing the formation of a narrow fusion pore, and events without projections (w/o proj.), representing non-measurable fusion pores in studied groups

	Con (47 cells)		Con + ATP (28 cells)		IFNγ (44 cells)		IFNγ + ATP (29 cells)	
	Count	%	Count	%	Count	%	Count	%
Exocytosis w proj	174	51 ± 5	88	45 ± 6	119	47 ± 6	94	64 ± 6
Exocytosis w/o proj	124	49 ± 5	80	55 ± 6	87	53 ± 6	66	36 ± 6
Total	298		168		206		160	
Endocytosis w proj	46	51 ± 7	41	45 ± 8	48	52 ± 7	28	48 ± 10
Endocytosis w/o proj	38	49 ± 7	47	55 ± 8	34	48 ± 7	28	52 ± 10
Total	84		88		82		56	

*Con* controls, *Con + ATP* non-treated controls after ATP stimulation,* IFNγ* IFNγ-treated astrocytes, *IFNγ + ATP* IFNγ-treated astrocytes after ATP stimulation

**Fig. 8:**
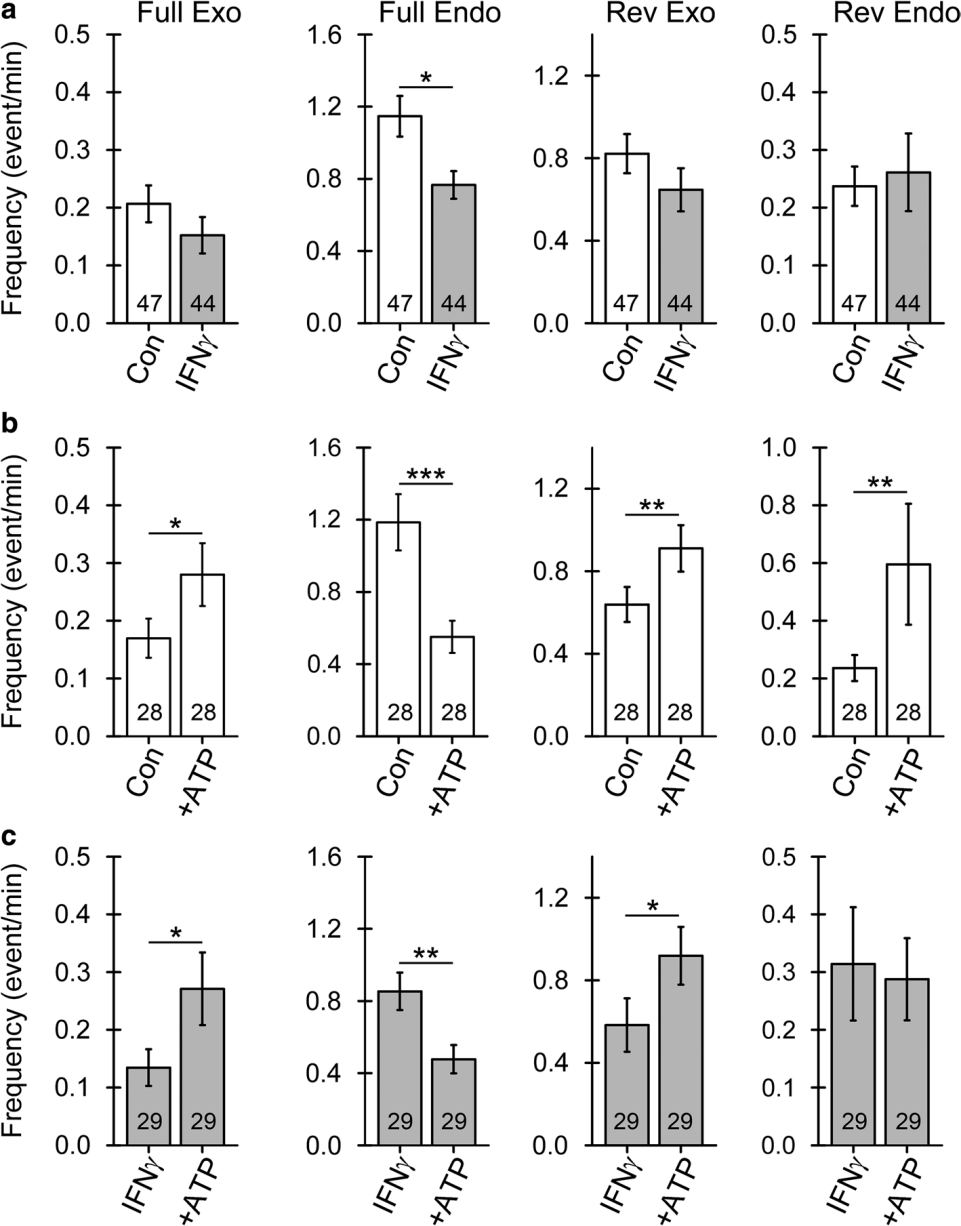
Astrocyte treatment by IFNγ affects the frequency of elementary exo- and endocytosis at rest and after ATP stimulation. **a** Frequency of elementary events (mean ± SEM) of full exocytosis (Full Exo), full endocytosis (Full Endo), reversible exocytosis (Rev Exo), and reversible endocytosis (Rev Endo) in controls and IFNγ-treated astrocytes. Note the decreased frequency of full endocytotic events in IFNγ-treated astrocytes. **b** and **c** Frequency of elementary events of exo- and endocytosis (as in **a**) before and after 100 µM ATP stimulation in controls (**b**) and IFNγ-treated astrocytes (**c**). Note that ATP stimulation similarly affects the frequency of exo- and endocytotic events in controls and IFNγ-treated astrocytes *P < 0.05, **P < 0.01, ***P < 0.001 (Wilcoxon signed-rank test). Numbers at the bottom of the bars indicate the number of cells analyzed

At rest, when cells were not stimulated with ATP, the frequency of reversible exo-/endocytotic events, as well as of full exocytotic events, was similar in non-treated and in IFNγ-treated cells (Fig. 8a). In contrast, full endocytotic events in IFNγ-treated astrocytes were less frequent than in controls (1.15 ± 0.11/min versus 0.77 ± 0.08/min, *p* < 0.05, respectively; Fig. 8a), indicating reduced vesicular uptake of material deriving from the extracellular space or plasmalemmal constituents. Notably, as the relative proportion of full endocytotic events in IFNγ-treated astrocytes decreased, the relative proportion of reversible endocytotic events increased, indicating a change in the process of endosome scission (Table 1).

### Astrocyte stimulation by ATP enhances exocytosis and inhibits endocytosis

Stimulation of control and IFNγ-treated astrocytes with 100 µM ATP to increase cytosolic calcium [53] led to an increase in the frequency of reversible (0.64 ± 0.09/min versus 0.91 ± 0.11/min, *p* < 0.05, and 0.58 ± 0.13/min versus 0.92 ± 0.14/min, *p* < 0.05, respectively) and full exocytotic events (0.17 ± 0.03/min versus 0.28 ± 0.05/min, *p* < 0.05, and 0.14 ± 0.03/min versus 0.27 ± 0.06/min, respectively), as well as a decrease in the frequency of full endocytotic events (1.19 ± 0.16/min versus 0.55 ± 0.09/min, *p* < 0.001, and 0.85 ± 0.10/min versus 0.48 ± 0.08/min, *p* < 0.01, respectively) (Fig. 8b, c). Stimulation of IFNγ-treated cells with ATP did not affect the frequency of reversible endocytotic events (0.31 ± 0.09/min versus 0.28 ± 0.07/min); however, this increased in non-treated controls (0.24 ± 0.04/min versus 0.59 ± 0.20/min, *p* < 0.05, respectively) (Fig. 8b, c). The diameter of vesicles undergoing reversible exocytosis was larger in astrocytes treated with IFNγ.

The relative proportion of reversible exocytotic events displaying a projection to the Re part of the admittance signal (i.e., present when the fusion pore is relatively narrow in diameter) in controls was similar before and after ATP stimulation (51 ± 5% versus 45 ± 6%, *p* = 0.42, respectively) as well as in IFNγ-treated astrocytes (47 ± 6% versus 64 ± 6%, *p* = 0.054, respectively). In reversible endocytotic vesicles, the relative proportion of vesicles reversibly interacting with the plasmalemma and exhibiting a narrow fusion pore remained similar in controls and in IFNγ-treated cells before and after ATP stimulation (51 ± 7% versus 45 ± 8%, *p* = 0.60, and 52 ± 7% versus 48 ± 10%, *p* = 0.86, for controls and IFNγ-treated cells respectively; Table 2). In control cells, the fusion pore conductance decreased significantly after ATP stimulation (117 ± 39 pS versus 53 ± 10 pS, *p* < 0.05) but it remained unchanged in IFNγ-treated cells (287 ± 82 pS versus 110 ± 20 pS, *p* = 0.35; Fig. 7a). The fusion pore dwell time in exocytotic vesicles was not affected by ATP stimulation in either group (Fig. 7b). In contrast, in endocytotic vesicles, the pore conductance in non-treated controls was not altered by ATP stimulation (86 ± 14 pS versus 67 ± 20 pS, *p* = 0.07), whereas it increased in ATP-stimulated IFNγ-treated cells (89 ± 18 pS versus 139 ± 26 pS, *p* = 0.050; Fig. 7a). In endocytotic vesicles, ATP stimulation evoked contrasting changes in fusion pore dwell time in both groups. In non-treated controls, the dwell time decreased from 0.5 ± 0.1 s to 0.2 ± 0.1 s (*p* < 0.05), whereas in IFNγ-treated cells, it increased from 0.3 ± 0.1 s to 0.6 ± 0.1 s (*p* < 0.05) after ATP stimulation (Fig. 7b).

### Astrocyte treatment with IFNγ reduces fluid-phase uptake

**Fig. 9:**
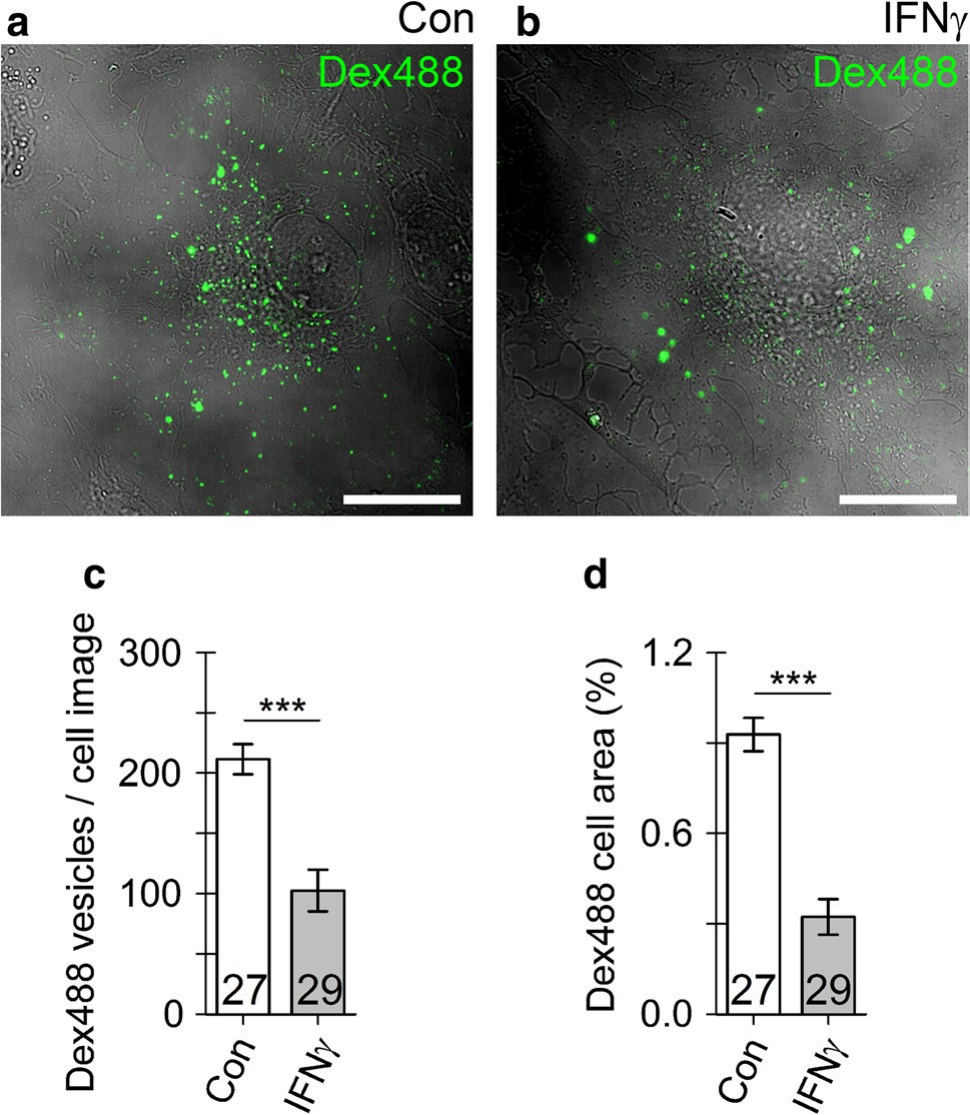
Fluid-phase endocytosis is suppressed in IFNγ-treated astrocytes. **a**, **b** Representative confocal images of an control astrocyte (**a**) and IFNγ-treated astrocytes (**b**) incubated for 3 h with 10 kDa dextran Alexa 488 conjugates (Dex488, green). Note individual Dex488-laden vesicles visible as bright fluorescent puncta (green). Scale bar: 20 µm. **c**, **d** Graphs displaying the number (mean ± SEM) of Dex488-laden vesicles per cell (**c**) and the relative proportion of Dex488-positive cell area normalized to cell image area (analogous to Fig. 1d) in controls (Con) and IFNγ-treated astrocytes. The numbers at the bottom of the bars indicate the number of cell images analyzed. ***P < 0.001 versus control (Mann–Whitney *U* test)

To optophysiologically study the modulation of astrocytic endocytic activity evoked by IFNγ, we examined the fluid-phase uptake of fluorescently labeled dextran (Dex488; Fig. 9). As opposed to more selective higher molecular weight dextran, 10 kDa dextran enters the cell via both micro- and macropinocytosis and subsequently traffics to lysosomes for degradation [54]. We found that IFNγ-treated astrocytes had a lower number of Dex488-positive endosomes (Fig. 9c) when compared to non-treated controls. Furthermore, IFNγ treatment reduced the amount of accumulated Dex488 molecules in astrocytes, as indicated by the percentage of cell area occupied by dextran fluorescence normalized to the whole cell area (Fig. 9d). Thus, IFNγ treatment reduces fluid-phase uptake in astrocytes.

## Discussion

In this study, we characterized subcellular compartmentalization, distribution, and surface localization of MHCII in astroglia activated by the pro-inflammatory cytokine IFNγ. For the first time, we directly demonstrated that cell treatment with IFNγ alters the fusion pore geometry and kinetics in vesicles reversibly interacting with the plasmalemma and favors reversible exocytosis of larger, lysosome-like vesicles accompanied by a simultaneous decrease of full vesicle endocytosis. All these events enable delivery and prolonged retention of MHCII molecules at the surface of astrocytes.

### IFNγ enhances lysosomal MHCII trafficking toward the astroglial surface

To specifically examine the intracellular compartmentalization of astroglial MHCII, we treated astrocytes with IFNγ to generate a pro-inflammatory phenotype characterized by increased MHCII expression [14–16, 55]. Fixed and live-cell immunocytochemistry confirmed vesicular localization of expressed MHCII (Figs. 1–4). Increased vesicular MHCII immunofluorescence detected in GFAP-positive hippocampal astrocytes in organotypic slices treated with IFNγ further confirmed that this pro-inflammatory cytokine evokes MHCII expression in situ (Fig. S1). Although earlier studies suggested astroglial MHCII localization within the late endo-/lysosomal system [16, 56], the precise identification of MHCII-positive compartments and their interaction with the plasmalemma remained unclear. Our study reveals that astroglial MHCII localizes only negligibly to early and recycling endosomes carrying Rab4A, EEA1, and TPC1 [24, 25] and modestly to compartments carrying Rab7, which regulates transport from early to late endolysosomes and plays a role in autophagosome maturation [24]. MHCII predominantly localized to LAMP1-EGFP-positive lysosomes (Fig. 2), which was further corroborated by pre-incubating IFNγ-treated astrocytes with GPN, a lysosomolytic agent [45, 46] that caused a marked reduction of MHCII immunofluorescence to levels observed in non-treated controls (Fig. 3). Concomitantly, the diameter of MHCII-positive vesicles estimated by super-resolution SIM corresponds well to the diameter determined in LAMP1-immunopositive astroglial vesicles [44]. MHCII thus principally resides in astroglial lysosomes; this pattern of subcellular localization is consistent with the one observed in immature dendritic cells, where up to 75% of MHCII is present in lysosome-like antigen-processing compartments, represented by late endo-/lysosomes enriched in proteolytic enzymes with a sufficiently low pH to activate proteases for antigen processing [57]. In addition, the relatively low acidity of astrocytic lysosomes preserves antigens for successful antigen presentation [58], again similarly to the effect observed in dendritic cells [59, 60]. Abundant surface immunolabeling of MHCII in non-permeabilized IFNγ-treated astrocytes (Fig. 4) confirmed successful incorporation of MHCII molecules into the plasmalemma and their prolonged exposure on the astrocyte surface. This finding is supported by previous flow cytometry [16] and immunoelectron microscopy results [56].

### Increased reversible exocytosis of larger vesicles indicates increased lysosomal fusion in IFNγ-treated astrocytes

Maturation of dendritic cells triggers the translocation of intracellularly stored peptide-loaded MHCII to the cellular surface. This action begins with tubulation of MHCII-containing late endo-/lysosomes toward the cell surface [61–63], followed by fusion of tubulated late endo-/lysosomes with the plasmalemma [62, 64]. MHCII-containing late endo-/lysosomes may fuse directly with the plasmalemma [62, 64], or alternatively, other vesicular intermediates responsible for plasmalemmal fusion could emanate, such as small MHCII-containing vesicles that bud from tubulated late endo-/lysosomes observed by electron microscopy in dendritic cells [65–67].

Because astrocytes actively communicate with their environment through exo-/endocytosis [68, 69], and lysosomes are equipped with the molecular machinery enabling membrane fusion and release of low-molecular-weight signaling molecules, they appear legitimate vesicular candidates to relocate MHCII to the plasmalemma. Lysosomal exocytosis can be either reversible or full, depending on the causative stimuli. The former is mostly associated with receptor stimulation, whereas the latter is predominately triggered by Ca^2+^ ionophores, potassium cyanide, mechanical stimulation, or plasmalemmal damage [39, 42, 70], and appears to be primarily involved in plasma membrane repair [70, 71]. In agreement with previous findings derived from membrane capacitance recordings, we observed full and reversible exocytosis [44, 50, 51, 72–74] with the latter predominating [44, 74]. Importantly, in IFNγ-treated astrocytes, the reversible exocytosis of larger vesicles indicates that the nature of vesicles undergoing transient exocytosis has changed (Fig. 6) and suggests increased involvement of lysosomes and larger exocytotic compartments in astroglia [69], whereas the nature of vesicles undergoing endocytosis remained similar in non-treated controls and IFNγ-treated astrocytes. Moreover, the diameters of exocytotic vesicles reversibly interacting with the plasmalemma (194 ± 10 nm; Fig. 6) largely correspond to the diameters of MHCII-positive vesicles in the periplasmalemmal space (230 ± 4 nm; Fig. 4) and the mean vesicle diameter of ~ 200 nm reported in immunolabeled LAMP1 [44], all indicating that newly emerged exocytotic compartments are astrocytic lysosomes that carry MHCII. In a previous study by Vardjan et al. [14], no release of fluorescent dextrans indicative of full lysosomal exocytosis was observed in IFNγ-treated astrocytes either at rest or after cell stimulation with 1 mM ATP [16], a stimulus that increases the intracellular concentration of free Ca^2+^ ions [Ca^2+^]_i_ [73, 75, 76]. This is likely explained by a relatively short post-stimulation time during which potential secretion from cells was monitored [16], because lysosomal exocytosis was shown to occur with a significant delay after cell stimulation [40, 41, 43].

Vesicles undergoing reversible exocytosis in IFNγ-treated astrocytes establish wider fusion pores with longer open pore dwell time (Fig. 7). These results not only confirm a positive correlation between vesicle capacitance and fusion pore conductance initially described in lactotrophs [52] but also suggest that cell treatment with IFNγ alters the molecular nature of exocytosis. In astroglial lysosomes, synaptotagmin XI (Syt XI) was identified as the regulator of the fusion pore dynamics. Silencing of Syt XI caused a threefold decrease in the frequency of lysosome exocytosis and a twofold increase in the number of reversible exocytotic events [70]. Modulation of either expression or function of Syt XI by IFNγ could well explain some of our findings. Secretory lysosomes in astrocytes also store and secret ATP [39, 40, 77, 78]. An increase in extracellular ATP concentration represents a danger signal that can lead to increased damage to neurons, promote reactive gliosis, and recruit microglia to instigate a neuroinflammatory response [79]. An increase in the occurrence of reversible fusion of larger vesicles may suggest increased leakage of lysosomal ATP to the extracellular space. Furthermore, the prolonged vesicle interaction with the plasmalemma may per se indicate an increased tendency toward full vesicle fusion, enabling complete incorporation of MHCII molecules into the astrocyte plasmalemma.

### In IFNγ-treated astroglia, endocytosis is downregulated, whereas ATP-evoked exocytosis remains intact

The predominant type of vesicle–plasmalemma interaction in resting astrocytes is full endocytosis, as reported previously [51]. Endocytotic vesicle retrieval, however, was significantly reduced in IFNγ-treated astrocytes, and fluid-phase uptake was markedly downregulated. These functional alterations may facilitate prolonged retention of MHCII on the cell surface and thus more effective antigen presentation. Similarly, during maturation of dendritic cells, modulation of endocytosis occurs, with downregulation of both macropinocytosis and phagocytosis [80, 81]. In macrophages, IFNγ treatment decreases macropinocytosis and mannose receptor-mediated uptake and promotes sorting of endosomes toward perinuclear lysosomes [82, 83]. Endocytosis thus plays an important role in regulating the distribution of MHCII in immune cells. Apparently, a similar mechanism operates in IFNγ-treated astrocytes.

As previously observed in cell-attached membrane capacitance measurements [44, 74], stimulation of astrocytes with ATP triggered an increase in the frequency of reversible and full vesicle exocytosis (Fig. 8). In addition, astrocyte stimulation with 1 mM ATP causes a net increase in bulk membrane capacitance [73]. Besides facilitating vesicle exocytosis, membrane surface area can also increase at the expense of diminished endocytosis, resulting in vesicle retention at the plasmalemma. Indeed, we observed robust downregulation of full endocytosis in controls and IFNγ-treated astrocytes stimulated with ATP, which has not been observed before in either astrocytes or other secretory cells. In contrast, an influx of Ca^2+^ is proposed as the universal trigger for all forms of vesicle endocytosis in secretory cells [84], and ATP acting on P2Y_4_ receptors augments macropinocytosis in microglia, where large vesicles with diameter ranging from 0.5 to 5 μm are formed [85]. However, relatively little is known about the endocytotic pathways in astrocytes. A constitutive endocytotic pathway regulated by intracellular Ca^2+^ concentration ([Ca^2+^]_i_) and independent of clathrin and caveolin was described in astrocytes in which endosomes were labeled with FM dyes [86]. However, it is contested whether FM dyes representatively label astrocyte endosomes [87]. A recent super-resolution microscopic visualization of fusion pores in chromaffin cells revealed an important role of increased [Ca^2+^]_i_ in constricting and closing these pores and thus regulating endocytosis [88]. However, [Ca^2+^]_i_ also plays an important role in triggering initial fusion pore opening and vesicle exocytosis in secretory cells. Conceivably, increased [Ca^2+^]_i_ facilitates both expansion and constriction of the fusion pore, yet at different concentrations, at lower and higher [Ca^2+^]_i_, respectively [84, 88]. Thus, the level of ATP-evoked increase in [Ca^2+^]_i_ could explain some of our observations.

In addition, inflammatory mediators and cytokines affect [Ca^2+^]_i_ responses in astrocytes [89, 90], potentially influencing vesicle exo-/endocytosis and thus affecting MHCII delivery and/or retention at the plasmalemma. Astrocyte activation with a cytokine cocktail containing IFNγ increases the expression of genes encoding G-protein-coupled receptors that contribute to [Ca^2+^]_i_ transients as well as other genes involved in Ca^2+^ signaling in astrocytes [90]. Reactive astrocytes located near β-amyloid plaques in an Alzheimer disease model displayed increased frequency of spontaneous [Ca^2+^]_i_ transients due to increased P2Y_1_ receptor signaling [91]. Nonetheless, ATP stimulation exerted a similar effect on vesicle exo-/endocytosis in IFNγ-treated and control astrocytes, with the sole exception of no increase in the frequency of reversible vesicle endocytosis in IFNγ-treated cells (Fig. 8). Despite some evidence that IFNγ directly affects [Ca^2+^]_i_ homeostasis in astrocytes [92], astrocyte treatment with IFNγ thus does not significantly alter the overall exo-/endocytotic response to ATP.

In conclusion, our study reveals novel insights into the dynamics of single-vesicle exo-/endocytosis altered by the inflammatory cytokine IFNγ, which augments the expression of MHCII in activated astrocytes [13–16]. We have demonstrated that MHCII expressed de novo predominantly sequesters into late endo-/lysosomes, which may undergo exocytosis [39–43] and deliver MHCII to the astrocyte surface. Cell treatment with IFNγ altered the nature of exocytotic vesicles interacting with the plasmalemma, whereby preferentially larger exocytotic vesicles deliver MHCII to the surface. Importantly, astrocytes treated with IFNγ displayed reduced endocytosis, which removes MHCII from the plasmalemma. Astrocyte treatment with IFNγ thus favors the synthesis and incorporation of de novo synthesized MHCII into the plasmalemma and prolongs MHCII retention at the surface, both potentially influencing antigen presentation to immune cells that may invade the brain parenchyma through the compromised blood–brain barrier [5]. Nonetheless, studies that examined glial MHCII expression in situ after administration of IFNγ to live animals found varying and delayed expression of MHCII on astrocytes [14, 93, 94]. The finding that MHCII expression in astrocytes is suppressed in vivo by neuronal activity [95] could explain some inconsistencies; however, further in vivo research is needed to determine the full pathophysiological impact of this process.

### Electronic supplementary material

Below is the link to the electronic supplementary material.

Supplementary file1 (PDF 350 kb)

## Abbreviations

BSA: Bovine serum albumin;
CNS: Central nervous system
DMEM: Dulbecco’s modified Eagle’s medium
GPN: Glycyl-l-phenylalanine 2-naphthylamide
IFNγ: Interferon γ
LyTr: LysoTracker red
MHCII: Major histocompatibility complex class II
PBS: Phosphate-buffered saline
RT: Room temperature
SIM: Structured illumination microscopy
Th: T helper
