# Supplementary material for: Exocytosis of large-diameter lysosomes mediates interferon γ-induced relocation of MHC class II molecules toward the surface of astrocytes
Source: Cell Mol Life Sci. 2019 Oct 30;77(16):3245–64. doi: 10.1007/s00018-019-03350-8 (PMC7391398; doi:10.1007/s00018-019-03350-8)

Supplementary material

Title: Exocytosis of large-diameter lysosomes mediate interferon γ-induced relocation of MHC class II molecules toward the surface of astrocytes

Authors: Mićo Božić[2,#], Alexei Verkhratsky[1,3,4,5], Robert Zorec[1,2,*], Matjaž Stenovec[1,2,#,*]

[1]Celica BIOMEDICAL, Tehnološki park 24, 1000 Ljubljana, Slovenia

[2]Laboratory of Neuroendocrinology-Molecular Cell Physiology, Institute of Pathophysiology, Faculty of Medicine, University of Ljubljana, Zaloška 4, 1000 Ljubljana, Slovenia

[3]Faculty of Biology, Medicine and Health, The University of Manchester, Manchester, M13 9PT, UK

[4]Achucarro Center for Neuroscience, IKERBASQUE, 48011 Bilbao, Spain

[5]Center for Basic and Translational Neuroscience, Faculty of Health and Medical Sciences, University of Copenhagen, Copenhagen 2200, Denmark

[#]Mićo Božić and Matjaž Stenovec contributed equally to this work.

*Correspondence: robert.zorec@mf.uni-lj.si, telephone: +386 1 543 70 80 (RZ); matjaz.stenovec@mf.uni-lj.si, telephone: +386 1 543 70 81 (MS).

Fig. S1 IFNγ treatment increases expression of MHCII in GFAP-positive hippocampal rat astrocytes in organotypic slices (a and b) Confocal images of control hippocampal astrocytes (Con, A) and astrocytes treated with IFNγ for 48 h (IFNγ, b), immunolabeled by anti-GFAP and secondary Alexa 488-conjugated antibody (green), and counterstained with DAPI (blue). Scale bars: 50 μm (a and b). (c) Confocal image of double-labeled astrocyte displaying immunofluorescent MHCII (red) and GFAP (green). Scale bar: 20 μm (large images; c–h) and 1 μm (insets; C, G, and H). (d–f) Confocal (d) and the corresponding thresholded mask images (e and f) of astrocyte GFAP fluorescence (the same as displayed in c) depict stages of estimation of the GFAP-positive cell area (for details, see Materials and

methods). (g and h) Immunofluorescent MHCII vesicles (red) in control (g) and IFNγ-activated astrocytes (h). The white curve outlines the cell (GFAP) area. Insets display a magnified view of the MHCII-positive vesicles in control and IFNγ-activated astrocytes. (i) The relative proportion of MHCII-positive cell area (%; surface area of MHCII-positive pixels with fluorescence above 20% of maximal fluorescence) normalized to cell (GFAP) area. Note the substantial increase in the proportion of MHCII-positive cell area in IFNγ-activated astrocytes. The numbers at the bottom of the bars indicate the number of cell images analyzed. ***$P < 0.001$ versus control (Mann-Whitney U test).

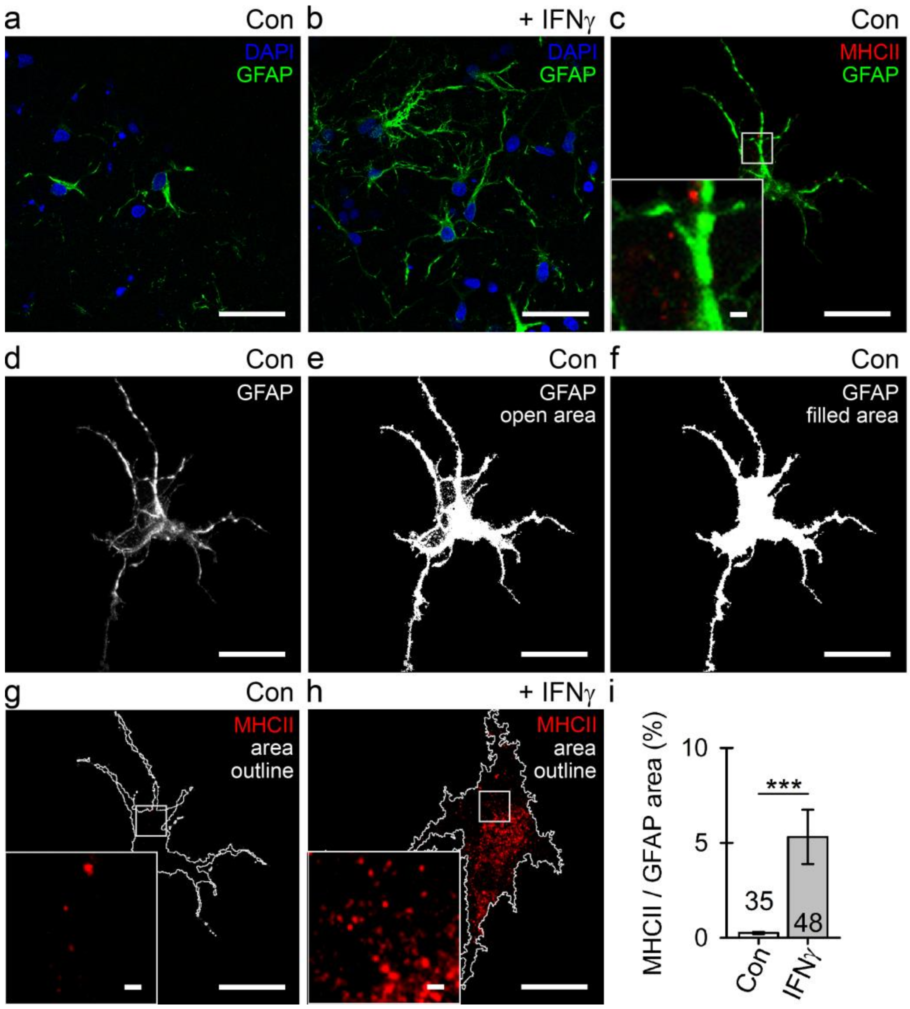

Supplement: Supplementary file 1 — Supplementary file1 (PDF 350 kb) [file 18_2019_3350_MOESM1_ESM.pdf]
